# Enhanced hyperalignment via spatial prior information

**DOI:** 10.1002/hbm.26170

**Published:** 2022-12-21

**Authors:** Angela Andreella, Livio Finos, Martin A. Lindquist

**Affiliations:** ^1^ Department of Economics Ca' Foscari University of Venice Venice Italy; ^2^ Department of Developmental Psychology and Socialization University of Padova Padova Italy; ^3^ Department of Biostatistics Johns Hopkins University Baltimore Maryland USA

**Keywords:** functional alignment, fMRI data, hyperalignment, Procrustes method, von Mises–Fisher distribution

## Abstract

Functional alignment between subjects is an important assumption of functional magnetic resonance imaging (fMRI) group‐level analysis. However, it is often violated in practice, even after alignment to a standard anatomical template. Hyperalignment, based on sequential Procrustes orthogonal transformations, has been proposed as a method of aligning shared functional information into a common high‐dimensional space and thereby improving inter‐subject analysis. Though successful, current hyperalignment algorithms have a number of shortcomings, including difficulties interpreting the transformations, a lack of uniqueness of the procedure, and difficulties performing whole‐brain analysis. To resolve these issues, we propose the ProMises (Procrustes von Mises–Fisher) model. We reformulate functional alignment as a statistical model and impose a prior distribution on the orthogonal parameters (the von Mises–Fisher distribution). This allows for the embedding of anatomical information into the estimation procedure by penalizing the contribution of spatially distant voxels when creating the shared functional high‐dimensional space. Importantly, the transformations, aligned images, and related results are all unique. In addition, the proposed method allows for efficient whole‐brain functional alignment. In simulations and application to data from four fMRI studies we find that ProMises improves inter‐subject classification in terms of between‐subject accuracy and interpretability compared to standard hyperalignment algorithms.

## Graphical abstract

Functional alignment is reformulated as a statistical model where prior anatomical information guides the estimation of orthogonal transformations. This allows us to resolve several outstanding issues related to hyperalignment, including interpretation, uniqueness, and whole‐brain application. The method improves the accuracy and interpretability of inter‐subject classification compared to standard hyperalignment methods.

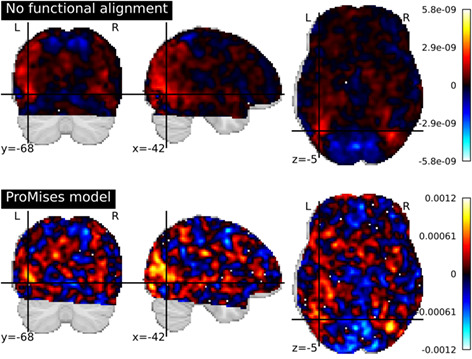

## 1 INTRODUCTION

Multi‐subject functional magnetic resonance imaging (fMRI) data analysis is important as it allows for the identification of shared cognitive characteristics across subjects. However, to be successful these analysis must properly account for individual brain differences. Indeed, it has been shown that the brains' anatomical and functional structures show great variability across subjects, even in response to identical sensory input (Hasson et al., 2004; Tootell et al., 1995; Watson et al., 1993). Various approaches have been proposed to deal with anatomical misalignment; these approaches align the images with a standard anatomical template (Fischl et al., 1999; Jenkinson et al., 2002; Talairach & Tournoux, 1988). However, these methods do not take into consideration the functional characteristics of the data; they fail to capture the shared functional response across subjects, ignoring the between‐subject variability in the anatomical positions of functional loci.

The problem of functional variability between subjects has long been known to neuroscientists (Hasson et al., 2004; Tootell et al., 1995; Watson et al., 1993). Indeed, this variation remains even after initial spatial normalization has been performed as a data preprocessing step. This can have serious consequences on group‐level fMRI analysis where it is generally assumed that voxel locations are consistent across subjects after anatomical alignment (Lindquist, 2008). A lack of functional alignment can lead to erroneous statistical inference, resulting in both power‐loss and reduced predictive accuracy (Wang et al., 2021). To address this issue, Haxby et al. (2011) proposed a functional alignment technique called hyperalignment, which uses orthogonal linear transformations to map brain images into a common abstract high‐dimensional space that represents a linear combination of each subjects' voxel activation profile. In practice, hyperalignment is a sequential application of the Procrustes transformation (Schonemmann & Carroll, 1970), which consists of finding the optimal rotation/reflection that minimizes the distance between subjects' activation profiles. The abstract high‐dimensional space created using hyperalignment represents the common information space across individuals as a mixture of overlapping, individual‐specific topographic basis functions (Haxby et al., 2020). Individual‐specific and shared functional information is modeled via high‐dimensional transformations rather than transformations that rely on three‐dimensional (3D) anatomical space. This innovative method of addressing the variability in the spatial location of functional loci across subjects has led to promising new research aimed at fostering an understanding of individual and shared cortical functional architectures (Conroy et al., 2009; Haxby et al., 2020).

Nevertheless, the approach has some shortcomings that remain to be addressed. First, hyperalignment remixes data across spatial loci (Haxby et al., 2011). Therefore, its use in aligning data from the entire cortex may be questionable because it combines information from distant voxels to create the common abstract high‐dimensional space (Haxby et al., 2020). This potentially undermines the ability to properly interpret the results. The method is powerful for classification; however, aligned images do not have a clear topographical interpretation. For this reason, hyperalignment is applied more appropriately to a region of interest (ROI). An alternative is searchlight hyperalignment (Guntupalli et al., 2016). Here overlapping transformations are calculated for overlapping searchlights in each subject and then aggregated into a single whole‐cortex transformation. This ensures that the voxels of the aligned images are generated from a circumscribed ROI, and thus allows for a topographical interpretation of the final map. However, this final transformation is no longer an orthogonal matrix, and therefore does not preserve the content of the original data, namely the similarity/dissimilarity in the response between pairs of voxels. In addition, the searchlights are imposed a priori and do not allow voxels outside of the predefined search radius to influence the estimation process. Second, as we show later in this work, the solutions calculated using hyperalignment are not unique in the sense that they depend directly on the order in which individuals are entered into the algorithm, as it is a sequential version of generalized Procrustes analysis (GPA) (Gower, 1975). Note that even the original GPA does not provide a unique solution. Third, while the idea of applying functional alignment to fMRI data is important, its application to whole‐brain analysis is problematic. In fact, most Procrustes‐based methods are based on singular value decomposition of square matrices whose dimensions equal the number of voxels. Therefore, it is infeasible to compute when working with the dimensions commonly used in whole‐brain fMRI studies.

This article proposes an approach that uses hyperalignment as a foundational principle, but at the same time resolves the aforementioned outstanding issues. The proposed method allows a researcher to decide how to combine individual responses to construct the common abstract high‐dimensional space where the shared functional information is represented. This is possible because the objective function of the Procrustes problem can be considered as a least‐squares problem. We reformulate it as a statistical model, which we denote the ProMises (Procrustes von Mises–Fisher) model. We assume a probability distribution for the error terms as well as a prior distribution for the orthogonal matrix parameter to restrict the range of possible transformations used to map the neural response into the common abstract high‐dimensional space. The constraint is based on specifics that the researcher has defined inside the hyperparameter of the prior distribution. We explain how to define this hyperparameter in an appropriate manner. Through a simple formulation, we show how the proposed model allows topographical information to be inserted into the estimation process and simultaneously computes unique solutions. Using the proposed model, researchers can move away from a black‐box approach, and instead better understand how functional alignment works by providing a neurophysiological interpretation of the aligned images as well as related results. In this way, the local radial constraints used in searchlight hyperalignment are surpassed. The model can incorporate them directly into the Procrustes estimation process, thus retaining all of hyperalignment's intrinsic properties, such as preserving the vector geometry.

The solution provides a unique representation of the aligned images and the related transformations (e.g., classifier coefficients, statistical tests, and correlations) in standardized anatomical brain space. In addition, the idiosyncratic topographies encoded inside the orthogonal transformation and the shared functional information do not depend on the specific reference matrix used by the algorithm. On the contrary, given that hyperalignment is a sequential approach of the Procrustes problem, the reference is not clear; it depends on the order of the subjects and the algorithm's successive steps. In our model, the coefficients forming the basis function of the common abstract high‐dimensional space are unique and reflect the orientation's prior information. Finally, to allow for whole‐brain analysis, we propose a computationally efficient version of the ProMises model, where proper semi‐orthogonal transformations project these square matrices into a lower‐dimensional space without loss of information.

The article is organized as follows. Subsection 2.1 outlines functional alignment via Procrustes‐based methods, while Subsection 2.2 describes some methods in the literature related to them, emphasizing their weaknesses. Thereafter, we offer a solution to these problems in Subsection 2.3, introducing the ProMises model as well as an efficient version of the model for whole‐brain analysis. Subsection 2.4 describes the four data sets explored. Finally, Section 3 illustrates the performance of the proposed alignment method within a multisubject classification framework. We compare the results with those obtained using no functional alignment (i.e., anatomical alignment only; Jenkinson et al., 2002) and functional alignment (after anatomical alignment) using GPA (Gower, 1975) and hyperalignment (Haxby et al., 2011).

## 2 METHODS

### 2.1 Functional alignment by Procrustes‐based method

The group neural activation can be described by a set of matrices, ${\left\{X_{i}\in {\mathbb{R}}^{t\times v}\right\}}_{i=1,\ldots ,m}$, one for each subject i. Here the t rows represent the response activation of v voxels at each time point, and the v columns represent the time series of activation for each voxel. The rows are ordered consistently across all subjects because the stimuli are time‐synchronized; however, the columns are not assumed to correspond across subjects (Hasson et al., 2004; Tootell et al., 1995; Watson et al., 1993). The functional alignment step is thus crucial for consistently comparing activation in a certain voxel between subjects (Haxby et al., 2020).

The most famous method for assessing the distance between matrices is the Procrustes transformation (Gower & Dijksterhuis, 2004). In simple terms, it uses similarity transformations (i.e., rotation and reflection) to match matrice(s) onto a target matrix as close as possible according to the Frobenius distance, using least‐squares techniques.

When one matrix, $X_{i}$, is transformed into the space of another $X_{j}$ via orthogonal transformation $R\in O\left(v\right)$, where $O\left(v\right)$ defines the set of orthogonal matrices in ${\mathbb{R}}^{v\times v}$, the Procrustes problem is called the orthogonal Procrustes problem (OPP):

(1) $$\min_{R\in O\left(v\right)}{\left\|X_{i}R-X_{j}\right\|}_{F}^{2},$$

where ${\left\|\cdot \right\|}_{F}$ denotes the Frobenius norm. The minimum is given by $R=UV^{⊤}$, where U and V come from the singular value decomposition (SVD) of $X_{i}^{⊤}X_{j}=\mathrm{U\sum}V^{⊤}$ (Schonemann, 1966).

Generally, fMRI group‐level analysis deal with m≥2 subjects. In this case, the functional alignment can be based on the GPA (Gower, 1975):

(2) $$\min_{R_{i}\in O\left(v\right)}\sum_{i=1}^{m}{\left\|X_{i}R_{i}-M\right\|}_{F}^{2},$$

where M is the element‐wise arithmetic mean of transformed matrices $X_{i}R_{i}$, also called the reference matrix. Equation 2 does not have a closed form solution, and is solved using an iterative procedure proposed by (Gower & Dijksterhuis, 2004). Alternatively, hyperalignment (Haxby et al., 2011) can be used, which is based on the sequential use of the OPP defined in Equation 1.

Importantly, both GPA and hyperalignment appear to have some shortcomings to resolve in order to yield unique, reproducible, and interpretable results. First, the orthogonal transformation $R_{i}$, computed via these methods, can combine information from every voxel inside of the cortical field or ROI. Anatomical structure is ignored, implicitly assuming that functional areas can incorporate neural activation of voxels from any part of the cortical area. A solution commonly used in the field is the searchlight approach proposed by (Guntupalli et al., 2016). However, this method assumes an optimal searchlight size, which must be defined by the researcher, thus introducing some degree of arbitrariness. Another approach which is more efficient than searchlight hyperalignment is to cluster voxels into sets of subregions across the whole brain as discussed by (Bazeille et al., 2021). In this way, the nonorthogonality problem of the searchlight hyperalignment approach is surpassed, but the set of subregions must again be defined a priori. In addition, in the parcel boundaries the optimality of this approach is not assured. Second, both methods return more than one solution (i.e., GPA has an infinite set of solutions, while hyperalignment has m! solutions, where m is the number of subjects). The $R_{i}$ computed via GPA are unique up to rotations. Instead, the $R_{i}$ calculated via hyperalignment strictly depends on the order of the subjects entering the algorithm. Being a sequential approach of the OPP, the choice of reference matrix is not clear, and every matrix used as a starting matrix leads to different common high‐dimensional spaces.

### 2.2 Hyperalignment‐related methods

After (Haxby et al., 2011), various modifications of hyperalignment have appeared in the literature. We do not list all the possible modifications here, and for a complete review please see (Bazeille et al., 2021; Cai et al., 2020). One of the most successful methods in the literature is the shared response model (SRM) proposed by Chen et al. (2015), which is a probabilistic model that computes a reduced dimension shared feature space. The method was also reformulated in matrix format by Shvartsman et al. (2018) and later analyzed by Cai et al. (2020) and Bazeille et al. (2021). In short, SRM estimates a semi‐orthogonal matrix with dimensions v×k, where k is a tunable hyper‐parameter representing the number of shared features. Therefore, as discussed by the authors, SRM returns a nonunique set of semi‐orthogonal transformations that leads to the loss of: (1) the original spatial characteristics; and (2) the topographical interpretation of the final aligned data. Similar to hyperalignment and GPA, SRM does not allow for the incorporation of spatial anatomical information into the estimation process, unlike the proposed ProMises model. Chen et al. (2015)'s method improves upon hyperalignment in terms of classification accuracy and scalability, but it analyzes the first k dimensions (i.e., latent variables), while hyperalignment is not constructed to be a dimensionality reduction technique. The scalability in Chen et al. (2015)'s method is improved as it requires the computation of singular values decompositions of matrices with smaller dimensions than those required by hyperalignment.

There are several promising functional alignment approaches in the literature that are not based on Procrustes theory, such as the optimal transport approach proposed by Bazeille et al. (2019). Comparisons with these methods would be interesting, but in this article we limit ourselves to analyzing Procrustes‐based approaches such as GPA and hyperalignment.

### 2.3 ProMises model

The focus of this article is to resolve the nonuniqueness and mixing problem of the transformations computed via GPA and hyperalignment. Indeed, solving these issues allows for the exploration of the structural neuroanatomy of functionally aligned matrices and their related transformations. The ProMises model resolves both points in an elegant and simple way, defining a hyper parameter for tuning the locality constraint. We stress here that it computes a unique solution that preserves the fine‐scale structure and allows for penalization of spatially distant voxels in the construction of the shared high‐dimensional space, assuming that the anatomical alignment is not too far from the central tendency.

To achieve these goals, we seek to insert prior information about the structure of $R_{i}$ into Equations (1), (2), which converts the set of possible orthogonal transformation solutions to a unique solution reflecting the prior information embedded. This is possible if we analyze the Procrustes problem from a statistical perspective. In short, the least squares problem formulate in Equations (1), (2) are reformulated as a statistical model, which allows for the definition of a prior distribution on $R_{i}$. To be precise, the difference between $X_{i}R_{i}$ and M described in Equation 2 can be viewed as an error term that is assumed to be normally distributed in our statistical model defined in the following subsection.

#### 2.3.1 Model

The minimization problem defined in Equation 2 can be reformulated as follows:

(3) $$\begin{array}{r} X_{i}=MR_{i}^{⊤}+E_{i}\;\text{subject to}\;R_{i}\in O\left(v\right), \end{array}$$

where $E_{i}\in {\mathbb{R}}^{t\times v}$ is the error matrix to minimize and $M\in {\mathbb{R}}^{t\times v}$ is the reference matrix.

We assume a multivariate normal matrix distribution (Gupta & Nagar, 2018) for the error terms $E_{i}$. Each row of $E_{i}$ is distributed as a multivariate normal distribution with mean 0 and covariance $\sum_{v}$. The observed data matrix $X_{i}$ is then described as a random Gaussian perturbation of M. The rotation matrix parameter $R_{i}$ allows for the representation of each data matrix $X_{i}$ in the shared functional space. In other words, the model simply reflects the assumption underlining hyperalignment, namely that neural activity in different brains are noisy rotations of a common space (Haxby et al., 2011). In this article, we assume $\sum_{v}=I_{v}$, where $I_{v}$ is the identity matrix of size v. The extension to an arbitrary type of variance matrix $\sum_{v}$ and incorporation of its estimation into the ProMises model is discussed in Andreella and Finos (2022).

#### 2.3.2 Prior information

Rephrasing the Procrustes problem as a statistical model allows us to impose a prior distribution on the orthogonal parameter $R_{i}$. With the constraint $R_{i}\in O\left(v\right)$ in equation 3, the probability distribution for $R_{i}$ must take values in the Stiefel manifold $V_{v}\left({\mathbb{R}}^{v}\right)$ (i.e., the set of all v‐dimensional orthogonal bases in ${\mathbb{R}}^{v}$). An attractive distribution on $V_{v}\left({\mathbb{R}}^{v}\right)$ is the matrix von Mises–Fisher distribution, introduced by Downs (1972) and further investigated by many others (Chikuse, 2003a, 2003b; Khatri & Mardia, 1977; Mardia et al., 2013; Prentice, 1986). It is defined as follows:

$$\begin{array}{r} f\left(R_{i}\right)=C\left(F,k\right)\exp\left\{\mathrm{Tr}\left(kF^{T}R_{i}\right)\right\}, \end{array}$$

where $\mathrm{Tr}\left(\cdot \right)$ defines the trace of a square matrix (i.e., the sum of elements on the main diagonal), $C\left(F,k\right)$ is a normalizing constant, $k\in {\mathbb{R}}_{\geq 0}$ is the concentration parameter, and $F\in {\mathbb{R}}^{v\times v}$ is the location matrix parameter.

The parameter k balances the amount of concentration of the distribution around F. As k→0, the prior distribution approaches a uniform distribution, representing the unconstrained case. In contrast, as k→+∞, the prior tends toward a distribution concentrated at a single point, representing the maximum constraint.

The polar part of F represents the mode of the distribution, and is unique if and only if F has full rank (Jupp & Mardia, 1976). In addition, the matrix von Mises–Fisher distribution is a conjugate prior (i.e., the posterior distribution has closed‐form expression in the same family of distributions as the prior) for the matrix normal distribution with posterior parameter equal to $X_{i}^{⊤}M+kF$. The solution for $R_{i}$ is unique if and only if $X_{i}^{⊤}M+kF$ has a full rank. Therefore, in the following, we define F such that it is of full rank and incorporates valuable information about the final high‐density common space.

The elements of the final high‐dimensional common space are composed of linear combinations of voxels (Haxby et al., 2020). Thus, F can be properly defined such that these combinations emphasize nearby voxels and penalize distant voxels. In this way, the anatomical structure of the cortex is used as prior information in the estimation of $R_{i}$. The idea is that nearby voxels should have similar rotation loadings, whereas voxels that are far apart should have less similar loadings. The hyperparameter F is defined as a Euclidean similarity matrix using the 3D anatomical coordinates of x, y, and z of each voxel:

(4) $$\begin{array}{r} F=\left[\exp\left\{-d_{\mathrm{ij}}\right\}\right]=\left[\exp\left\{-\sqrt{{\left(x_{i}-x_{j}\right)}^{2}+{\left(y_{i}-y_{j}\right)}^{2}+{\left(z_{i}-z_{j}\right)}^{2}}\right\}\right], \end{array}$$

where i,j=1,…v. In this way, F is a symmetric matrix with ones in the diagonal, which means that voxels with the same spatial location are combined with weights equal to 1, and the weights decrease as the voxels to be combined become more spatially distant.

F can also be specified via geodesic distances to exploit the intrinsic brain curve structure, or via the Dijkstra distance (Dijkstra, 1959) if surface‐based data are analyzed. In addition, another type of Minkowski measure (Upton & Cook, 2014) may be used; however, it is important to carefully consider the type of spatial information available, that is, the distance used must be reasonable in the context of the data. In this case, the Euclidean similarity matrix is an attractive measure for detecting how close the voxels overlap. In addition, F defined as in Equation 4, has full rank, which is a necessary condition for having a unique solution for $R_{i}$. The graphical representations of the Euclidean distance matrix using the 3D anatomical coordinates of voxels (or vertices of a surface grid) are proposed in the next section analyzing two types of data sets (face and object recognition and movie watching).

In summary, the ProMises model returns a solution that is a slight modification of the OPP solution that (Schonemann, 1966) developed for the case of two subjects, as well as a slight modification of the GPA solution that (Gower, 1975) proposed for the case of multiple subjects. The modification is based on applying the SVD to $X_{i}^{⊤}M+kF$ instead of $X_{i}^{⊤}M$. Thus, prior information about $R_{i}$ enters the SVD step through the specification of F, with the term k balancing the relative contribution of $X_{i}^{⊤}M$ and F. Thanks to this regularization, the ProMises model returns a set of unique transformations that correspond to the anatomical brain structure, exploiting the spatial location of voxels in the brain, or ROI. Hence, the ability to define the parameter F guarantees a topographical interpretation of the results, as we will see in the next section.

#### 2.3.3 Efficient ProMises model

The ProMises model returns a unique orthogonal transformation for each subject; however, it cannot be applied to the entire brain due to the extensive computational burden. This is due to the fact that at each step we must compute m singular value decompositions of v×v matrices leading to polynomial time complexity.

To allow for whole‐brain analysis, we propose the Efficient ProMises model, which allows for a faster functional alignment without loss of information. In practice, the Efficient ProMises model projects matrices $X_{i}$ into a t lower‐dimensional space via specific semi‐orthogonal transformations $Q_{i}\in {\mathbb{R}}^{v\times t}$ (Abadir & Magnus, 2005; Groß et al., 1999) which preserve all of the information in the data. It aligns the reduced t×t matrices ${\left\{X_{i}Q_{i}\in {\mathbb{R}}^{t\times t}\right\}}_{i=1,\ldots ,m}$, and back‐projects the aligned matrices to the original t×v‐size matrices ${\left\{X_{i}\in {\mathbb{R}}^{t\times v}\right\}}_{i=1,\ldots ,m}$ using the transpose of these semi‐orthogonal transformations ($Q_{i}^{⊤}\in {\mathbb{R}}^{t\times v}$).

No loss of information occurs because the minimum of Equation 2 using ${\left\{X_{i}Q_{i}\in {\mathbb{R}}^{t\times t}\right\}}_{i=1,\ldots ,m}$ is equivalent to the one obtained using the original data. This is due to the fact that the Procrustes problem analyzes the first *t* × *t* dimensions of $R_{i}$. Hence, the minimum remains the same if we use as our semi‐orthogonal matrices ${\left\{Q_{i}\right\}}_{i=1,\ldots ,m}$ the ones obtained from the thin singular value decomposition of ${\left\{X_{i}\in {\mathbb{R}}^{t\times v}\right\}}_{i=1,\ldots ,m}$.

The algorithms describing the ProMises model estimation process and its Efficient version are reported in Appendix 1. For further details and proofs about the ProMises model and its efficient version, please see Andreella and Finos (2022).

### 2.4 fMRI data sets

The performance of the proposed method is assessed using two fMRI data sets from Haxby et al. (2011) and one from Haxby et al. (2001) summarized in Table 1. We analyzed an additional data set collected by (Duncan et al., 2009). The data sets differ in several key characteristics, including number of subjects, whether data is extracted from an ROI or the whole‐brain, and the number of time points, voxels and stimuli. In addition, they differ depending on whether the data is in volumetric space or on a surface. These differences will allow us to evaluate the performance of the proposed model in a number of different circumstances.

**TABLE 1:** Description of the data sets used in our analysis.

Dataset	Subjects	ROI	Length	Voxels	Stimuli
Faces and objects	10	Ventral temporal cortex	56	3509	8
Visual object recognition	6	Whole brain	121	39,912	8
Raiders	31	Ventral temporal cortex	2662	883	400
Raiders	31	Occipital lobe	2662	653	400
Raiders	31	Early visual cortex	2662	484	400
Words and objects	12	Whole brain	164	73,574	5

*Note*: They differ in factors such as number of subjects, the region of interest, as well as the number of time points, voxels, and stimuli.

**FIGURE 1:**
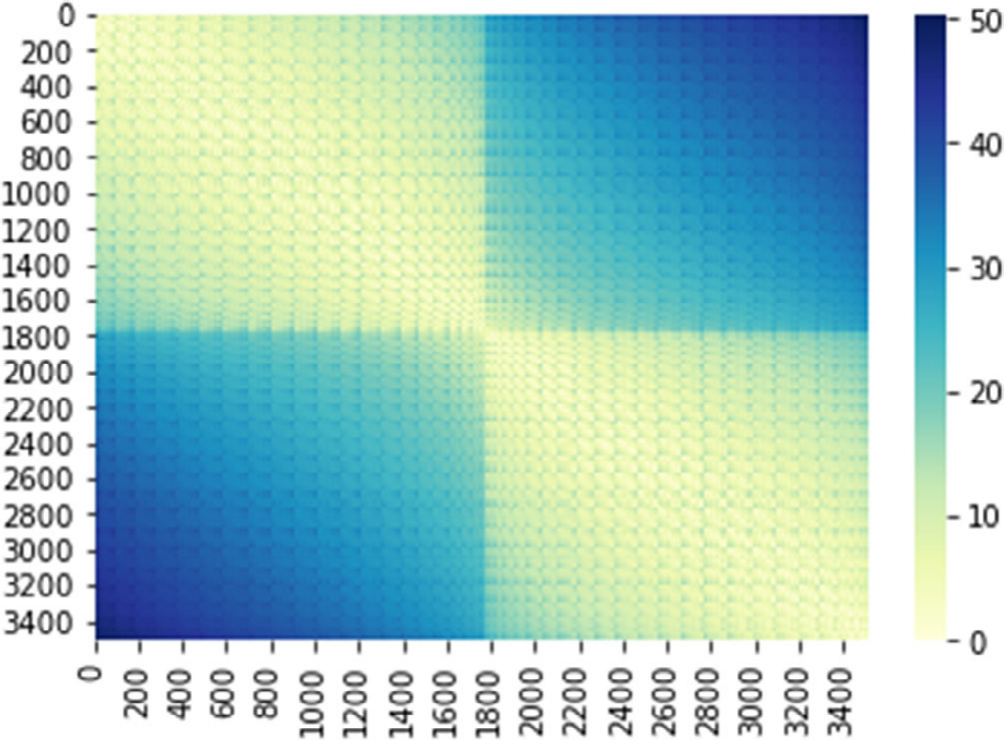
Representation of the Euclidean distance matrix to compute location parameter *F* of the von Mises–Fisher distribution using the 3D coordinates of the voxels from the faces and objects data set.

The first data set, referred to as *faces and objects*, is a block‐design fMRI study aimed at analyzing face and object representations in the human ventral temporal (VT) cortex. It is composed of fMRI images of 10 subjects with eight runs per subject. In each run, subjects look at static, gray‐scale images of faces and objects (i.e., human females, human males, monkeys, dogs, houses, chairs, and shoes). The subject views these images for 500 ms with 1500 ms inter‐stimulus intervals. Each block consists of viewing 16 images from one category, corresponding to a one‐back repetition detection task for each subject. Blank intervals of 12 divide the blocks. Each run contains one block of each stimulus category. Brain images were acquired using a 3T Siemens Allegra scanner with a standard bird‐cage head coil. Whole brain volumes of 32 3‐mm thick axial slices (TR = 2 s, TE = 30 ms, flip angle = 90°, 64 × 64 matrix, FOV = 192 mm × 192 mm) were obtained that included all of the occipital and temporal lobes and all but the most dorsal parts of the frontal and parietal lobes. High resolution T1‐weighted images of the entire brain were obtained in each imaging session (MPRAGE, TR = 2.5 s, TE = 4.3 ms, flip angle = 8°, 256 × 256 matrix, FOV = 256 mm × 256 mm, 172 1‐mm thick sagittal images). For further details about the experimental design and data acquisition, please see Haxby et al. (2011). Here, the analysis is focused on the 3509 voxels within the VT cortex. Figure 1 shows the Euclidean distance matrix used to calculate the location matrix parameter F of the von Mises–Fisher distribution for this data set. In this analysis, we define F as a Euclidean similarity matrix; see Equation 4. The two visible blocks represent the left and right VT cortex. A jump of four units (i.e., voxel index ijk units) in the *i*th dimension exists between voxel 1782 and voxel 1783, corresponding to the corpus callosum.

The second data set, referred to as visual object recognition has a similar structure as the faces and objects data set, where six subjects are viewing images of faces, cats, five categories of man‐made objects, and nonsense pictures for 500 ms with an inter‐stimulus interval of 1500 ms. Brain images were acquired on a GE 3T scanner (General Electric, Milwaukee, WI). Whole brain volumes of 40 3.5‐mm thick sagittal images (TR = 2500 ms, TE = 30 ms, flip angle = 90°, FOV = 24 cm) were obtained. High‐resolution T1‐weighted spoiled gradient recall (SPGR) images were obtained for each subject to provide detailed anatomy (124 1.2‐mm thick sagittal images, FOV = 24 cm). For further details, see Haxby et al. (2001). Here the analysis is focused on the use of whole‐brain data consisting of 39,912 voxels. Having a large number of voxels, we here use the Efficient ProMises model to align the brain images. In this case, the location matrix parameter is a lower‐dimensional version of $F\in {\mathbb{R}}^{v\times v}$, that is, the similarity Euclidean matrix defined in Equation 4. This new location matrix parameter must take values in ${\mathbb{R}}^{t\times t}$. It is expressed as $Q_{i}^{⊤}FQ_{M}$, where $Q_{i}$ is the semi‐orthogonal matrix coming from the thin singular value decomposition of $X_{i}$, and $Q_{M}$ is the semi‐orthogonal matrix coming from the thin singular value decomposition of M.

**FIGURE 2:**
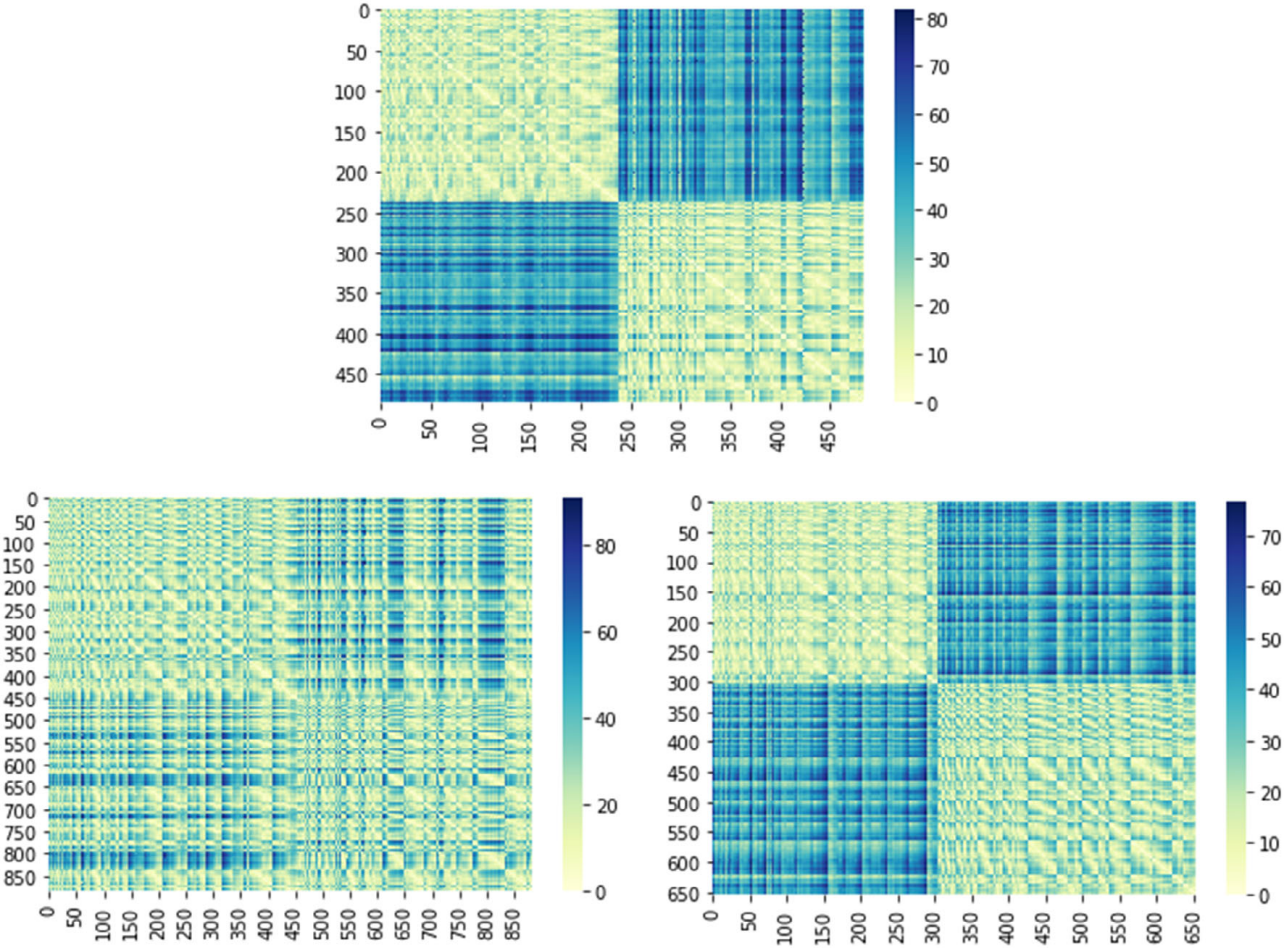
Representation of the Euclidean distance matrix used to compute the location parameter *F* of the von Mises–Fisher distribution using the 3D coordinates of the voxels from the *raiders* data set (Top: EV, bottom left: VT, bottom right: LO).

The third data set, referred to as *raiders*, consists of 31 subjects watching the movie “Raiders of the Lost Ark” (1981). The movie session was split into eight parts of approximately 14 min. Brain images were acquired using a 3 T Philips Intera Achieva scanner with an eight‐channel head coil. Brain volumes were obtained consisting of 41 3‐mm thick sagittal images (*R* = 2.5 s, TE = 35 ms, flip angle = 90°, 80 × 80 matrix, FOV = 240 mm × 240 mm). High resolution T1‐weighted images of the entire brain were obtained in each imaging session (MPRAGE, TR = 9.85 s, TE = 4.53 ms, flip angle = 8°, 256 × 256 matrix, FOV = 240 mm × 240 mm, 160 1‐mm thick sagittal images). For more details about subjects, MRI scanning parameters, data preprocessing, and ROI definition, see Haxby et al. (2011). Here the analysis is focused on ROIs in the VT cortex (883 voxels), occipital lobe (LO; 653 voxels), and early visual (EV; 484 voxels) cortex. Figure 2 shows the Euclidean distance matrix using the 3D coordinates from the ROIS defined over the VT cortex, LO, and EV cortex. The two blocks represent the left and right parts of the ROIs. In this case, the 3D coordinates describe the vertices of a surface grid based on the cortex envelope. The mapping from the volume to the surface was computed using the FreeSurfer software (Fischl et al., 1999). As in the first analysis (i.e., *faces and objects*), we defined the location matrix as a Euclidean similarity matrix as seen in Equation 4. However, in this case, the 3D coordinates refer to the vertices of the surface grid, as mentioned before. Thus, we could have defined F using geodesic distances. However, we found no substantial improvement in the results. Therefore, we prefer to use Euclidean distance since it provides a full‐rank matrix (i.e., a necessary property to achieve the uniqueness of the solution).

The fourth data set, referred to as words and objects, is a block‐design fMRI study to analyze brain regions, such as occipital temporal cortex, associated with functional word and object processing. In this study, 49 subjects view images of written words, objects, scrambled objects, and consonant letter strings for 350 ms with a 650 ms fixation cross at the beginning of each trial. The functional data were acquired with a gradient‐echo EPI sequence (TR = 3000 ms; TE = 50 ms; FOV = 192 × 192; matrix = 64 × 64) giving a resolution of 3×3×3 mm. A high‐resolution anatomical scan was acquired (T1‐weighted FLASH, TR = 12 ms; TE = 5.6 ms; 1 mm^3^ resolution) for each subject. For further details, see Duncan et al. (2009). As in the *visual object recognition* data analysis, we apply the Efficient ProMises model to align the whole brain image composed of 73,574 voxels.

For all analyses, we consider the set $K=\left\{1,2,\ldots ,100\right\}$ as a collection of possible values for the concentration parameter k. The optimal value is estimated by cross‐validation as explained in the next section.

The aim is to classify stimulus‐driven response patterns in a left‐out subject based on response patterns in other subjects. These patterns are described via a sequence of voxels that might express an activation at a specific time point. It is a vector in a high‐dimensional space, where each dimension represents a local feature (i.e., a voxel). Using multivariate pattern classification (MVPC) (Haxby, 2012), the patterns of neural activities are then classified by analyzing their variability during different stimuli (Kriegeskorte et al., 2008; O'Toole et al., 2007). We clarify here that the registration to standard MNI space is part of the preprocessing step. So, all functional alignment approaches are applied after spatial alignment to MNI space. We then evaluate the ProMises model in terms of across‐subject decoding accuracy (Bazeille et al., 2021) and interpretation of the final aligned images. We stress here that (Bazeille et al., 2021) found that SRM (Chen et al., 2015) and optimal transport (Bazeille et al., 2019) outperformed hyperalignment and searchlight hyperalignment (Guntupalli et al., 2016) at the ROI level. However, our aim is to provide a clear statistical model for functional alignment that permits one to incorporate spatial anatomical information into the estimation process, thereby leading to an optimal unique orthogonal transformation rather than focus on improving the classification predictive accuracy. The ProMises model proposed is compared in terms of between‐subjects predictive accuracy with GPA, and hyperalignment methods as well as anatomical alignment. We did not consider other related approaches (e.g., SRM, Chen et al., 2015; optimal transport, Bazeille et al., 2019) since we decided to focus on Procrustes‐based approaches (i.e., those that minimize an objective function with an orthogonality constraint) and to show the related variability of these approaches. For a complete review of functional alignment methods, please refer to Bazeille et al. (2021) and Cai et al. (2020).

We have developed a Python (Van Rossum & Drake Jr, 1995) module—ProMisesModel—available at https://github.com/angeella/ProMisesModel in line with the Python PyMVPA (Hanke et al., 2009) package. We also have created the alignProMises R (R Core Team, 2018) package available at https://github.com/angeella/alignProMises based on the C++ language.

## 3 RESULTS

### 3.1 Faces and objects

The protocol for evaluating the performance of the ProMises model directly follows the one used in (Haxby et al., 2011). We classify the patterns of neural activation using a support vector machine (SVM) (Vapnik, 1999). The between‐subject classification is computed using leave‐one‐out subject cross‐validation. To avoid the circularity problem (Kriegeskorte et al., 2009), the alignment parameters and the regularization parameter k (i.e., the concentration parameter) are fitted in the leave‐one‐out run using a nested cross‐validation approach. The performance metric used is the mean accuracy over leave‐one‐out subjects and leave‐one‐out runs. Note for each of the methods compared the input data is spatially normalized to MNI space (Jenkinson et al., 2002).

**FIGURE 3:**
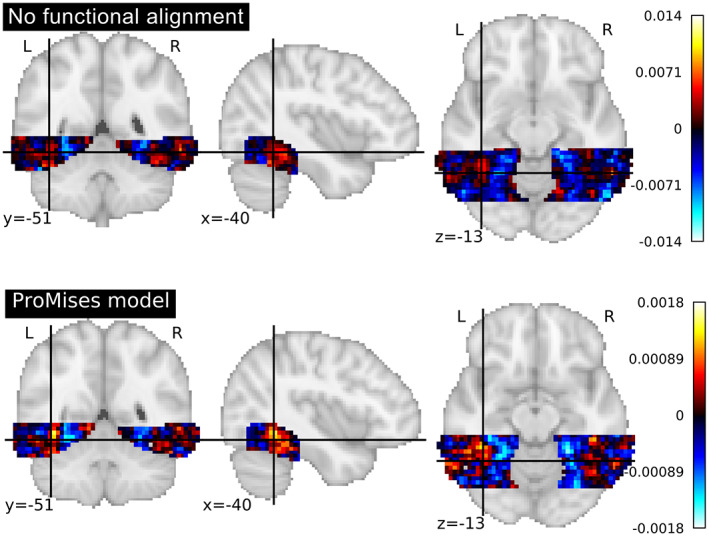
Coefficients of the multi‐class linear SVM considering the monkey face versus the male face classifier (where hot colors correspond to predicting male face) analyzing data aligned and not aligned via the ProMises model.

We perform classification using the full set of voxels (3509), and plot the classifier coefficients in the brain space. With seven class categories and a one‐versus‐one strategy (Lorena et al., 2008), 21 binary classifiers were fit. Figure 3 represents the coefficients of the monkey face versus the male face classifier. The plots representing the coefficients of the classification of fine‐grained distinctions in the object category and the coarse‐grained distinctions between categories are shown in Appendix 2. In Figure 3, we see that the coefficients of the classifier fit using anatomical alignment only is more diffuse than the equivalent values obtained using the ProMises model which appears to better capture the VT cortex's spatial anatomical geometry, as well as improve the ability to distinguish between categories. The between‐subjects accuracy equals 0.5 using no functional alignment, while it equals 0.7 using the ProMises model.

The computation time equals 57.109 s if no functional alignment is applied to the data, while it equals 1619.835 when using the ProMises model.

### 3.2 Visual object recognition

**FIGURE 4:**
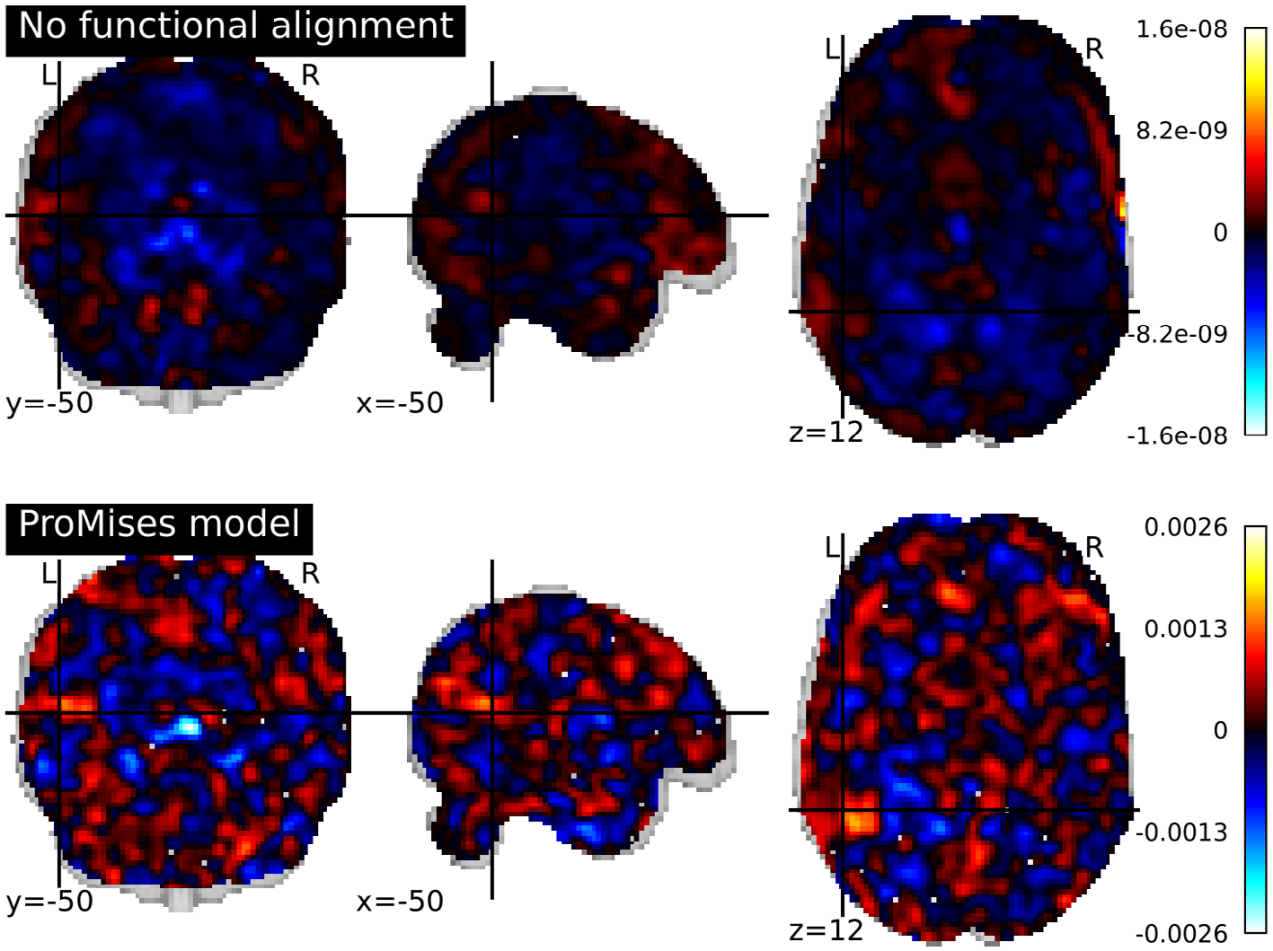
Coefficients of the multi‐class linear SVM considering the house versus face classifier (where hot colors correspond to predicting human face) analyzing data aligned and not aligned via the ProMises model.

For this data set the entire brain is functionally aligned using the Efficient ProMises model and classified via the SVM using the same process described for the faces and objects data set. Figure 4 represents the coefficients of the houses versus faces classifier using anatomically aligned only (Jenkinson et al., 2002) (top figure) and anatomically + functionally aligned (bottom figure) data. The Efficient ProMises model allows for the application of the classification to data from the entire brain, returning a between‐subject accuracy equal to 0.6, as well as a clear and interpretable brain map. In contrast, the anatomical alignment returns a more diffuse image with a between‐subject accuracy equal to 0.4.

**FIGURE 5:**
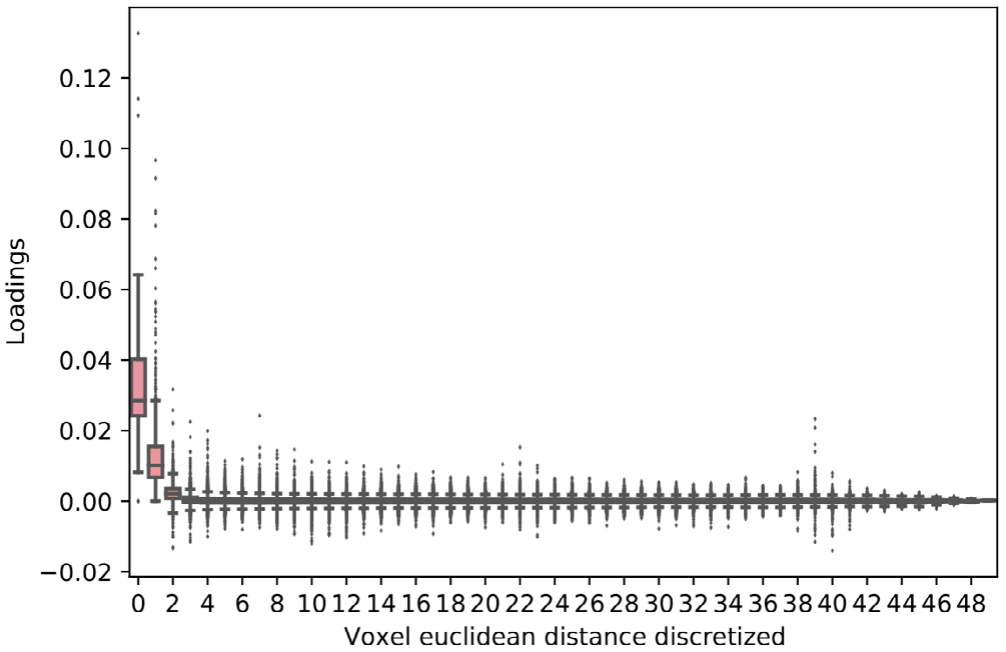
Boxplots of rotation loadings for each discretized value of the Euclidean distance between 50 voxels (randomly sampled) computed considering the 3D voxel indices *ijk* of the voxels. See the text for a detailed description of this figure.

One might think that whole‐brain functional alignment is not recommended because idiosyncratic functional‐anatomical correspondence generally occurs locally. The alignment must also avoid aligning different functional regions, such as the ventral temporal cortex of one subject with the prefrontal cortex in another subject. However, the Efficient ProMises model returns rotation coefficients that take into account the spatial brain information thanks to the specification of the prior distribution for the orthogonal parameters. These coefficients have high values for neighboring voxels and low values for distant voxels. For the visual object recognition data set, this result is shown in Figure 5 where the distribution of the loadings (i.e., contribution of the given voxel in the construction of the new, aligned, voxel) is shown as a function of the Euclidean distance (3D voxel indices ijk) of the original voxels to the new voxel. For visualization purposes, the boxplots are grouped by the discretized value of the Euclidean distances. To clarify, let us consider as an example the first element $x_{11i}$ of the (nonaligned) matrix $X_{i}$, and the first element ${\hat{x}}_{11i}$ of the aligned matrix ${\hat{X}}_{i}$, where ${\hat{x}}_{11i}=x_{11i}r_{11i}+x_{12i}r_{21i}+\ldots +x_{1\mathrm{mi}}r_{m1i}$ and $r_{\mathrm{kji}}\in Q_{i}{\hat{R}}_{i}Q_{i}^{⊤}$. In Figure 5, the voxel $x_{11i}$ will have a distance equal to 0 (in the abscissa), while the ordinate will be given by the value of the loading $r_{11i}$; other voxels $x_{12i},\ldots ,x_{1\mathrm{mi}}$ will have larger distances. Figure 5 shows that ProMises penalizes the combination of spatially distant voxels (i.e., loadings with small values) and prioritizes the combination of neighboring voxels (i.e., loadings with high values) in creating the new common abstract high‐dimensional space. To further support this claim we report that the median cumulative proportion of squared loadings is 50% at a distance of 19 voxels and is 90% at a distance of 37. Note that, indeed, a distance of 19 voxels is considerable. However, it is still clear that the ProMises model takes into account the spatial anatomical information of the voxels, although not stringently. If one wanted more spatial constriction, one could use a different location matrix (e.g., the identity matrix which gives weights to the voxel sharing the same coordinates) or the concentration parameter k. Thus, we claim that the efficient ProMises model returns linear transformations for the whole brain that also act locally. The computation time equals 3554.375 seconds if the ProMises model is used, while it equals 650.568 seconds if no functional alignment is applied to the data.

### 3.3 Raiders

The voxel responses are from the VT, LO, and EV ROIs, which are essential brain regions for analyzing the subject's reaction to visual stimuli, such as watching a movie. The alignment and regularization parameter k are computed using half of the movie and nested cross‐validation, and the between‐subject classification is performed on the remaining half to avoid circularity problems. The one nearest neighbors algorithm is used to classify the correlation vector composed of six time points (18‐s segment of the movie). The classification is correct when the correlation of the subject response vector with the group mean response vector (computed in the remaining subjects) is greater than the correlation between that vector response and the average response is to all other time segments. The classification is repeated for all one hold‐out subjects, and the average accuracy is computed as a performance metric.

The performance of the classification is tested using that was been anatomically aligned only, and data that is also functionally aligned using hyperalignment, GPA, and the ProMises model. As we can see in Table 2, the ProMises model returns a higher mean accuracy than when only using anatomical alignment. The improvement in between‐subjects accuracy using the proposed method is consistent across different ROIs. In addition, the between‐subjects accuracy is roughly twice as high as that obtained when not using any functional alignment.

**TABLE 2:** Classification accuracy for the raiders data set using the anatomical alignment, as well as ProMises alignment for three different ROIs (VT, EV, and LO).

	ROIs		
	VT	EV	LO
No functional alignment	0.289	0.534	0.238
ProMises model	0.472	0.709	0.568

**FIGURE 6:**
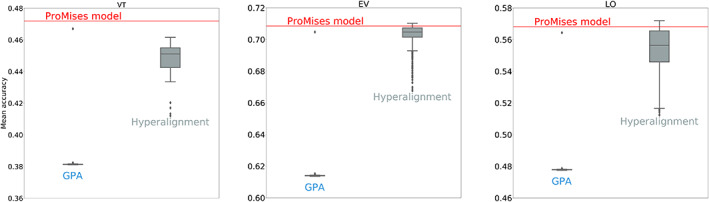
Boxplots representing the mean classification accuracy for the *raiders* data aligned using GPA and hyperalignment for three different ROIs (VT, EV, and LO). The results obtained using the ProMises model are shown as a red line.

It is important to note that various authors have already demonstrated this improvement in terms of classification accuracy using functional alignment as opposed to anatomical alignment (Haxby et al., 2011). However, here, we want to represent the variability of the between‐subject accuracy if hyperalignment or GPA are used instead of the ProMises model for functional alignment. In Figure 6, the gray boxplots represent the mean accuracy using hyperalignment, having permuted the order of the subjects in the data set 100 times. In contrast, the blue boxplots show the mean accuracy of GPA using 100 random rotations of the reference matrix M. Clearly, neither of the two methods return a unique solution of $R_{i}$, resulting in variability in the final classification results and complicating their interpretation. In contrast, the ProMises model provides a unique solution across all permutations, depicted as a red line in the figure.

Analyzing the hyperalignment results, the SD of the between‐subjects accuracy equals 0.01 for the VT analysis, 0.0141 for the LO analysis, and 0.0081 for the EV analysis. In contrast, using GPA the SD equals 0.014 for VT, 0.01 for LO, and 0.0078 for EV. For all three ROIs, the accuracy obtained using the ProMises model is generally higher than the maximum values obtained using GPA. For hyperalignment, the maximum value is higher than the results obtained using ProMises in EV and LO. We also applied the regularized hyperalignment approach proposed by Xu et al. (2012); however, we found that the performance results are optimal with a regularized parameter equal to 1 in all three frameworks (i.e., the Xu et al. (2012)'s method collapses to the standard hyperalignment case).

In the previous example, we empirically proved the nonuniqueness of hyperalignment and GPA. For a formal proof, see Andreella & Finos (2022). This result means that we have a different representation of the aligned images and related results in the brain space for each set of transformations. However, using the ProMises model, this can be avoided.

Computation times are reported in Table 3 for each analysis.

**TABLE 3:** Computation time (in seconds) for each analysis performed on the *raiders* data set, using a 3000+ core Linux cluster with 20 GB of random‐access memory

	VT	EV	LO
No functional alignment	234.32	204.798	188.003
ProMises model	7581.615	7003.431	7423.76
Hyperalignment	2728.47	2102.53	2481.74
GPA	48847.36	41748.33	47890.32

### 3.4 Words and objects

**FIGURE 7:**
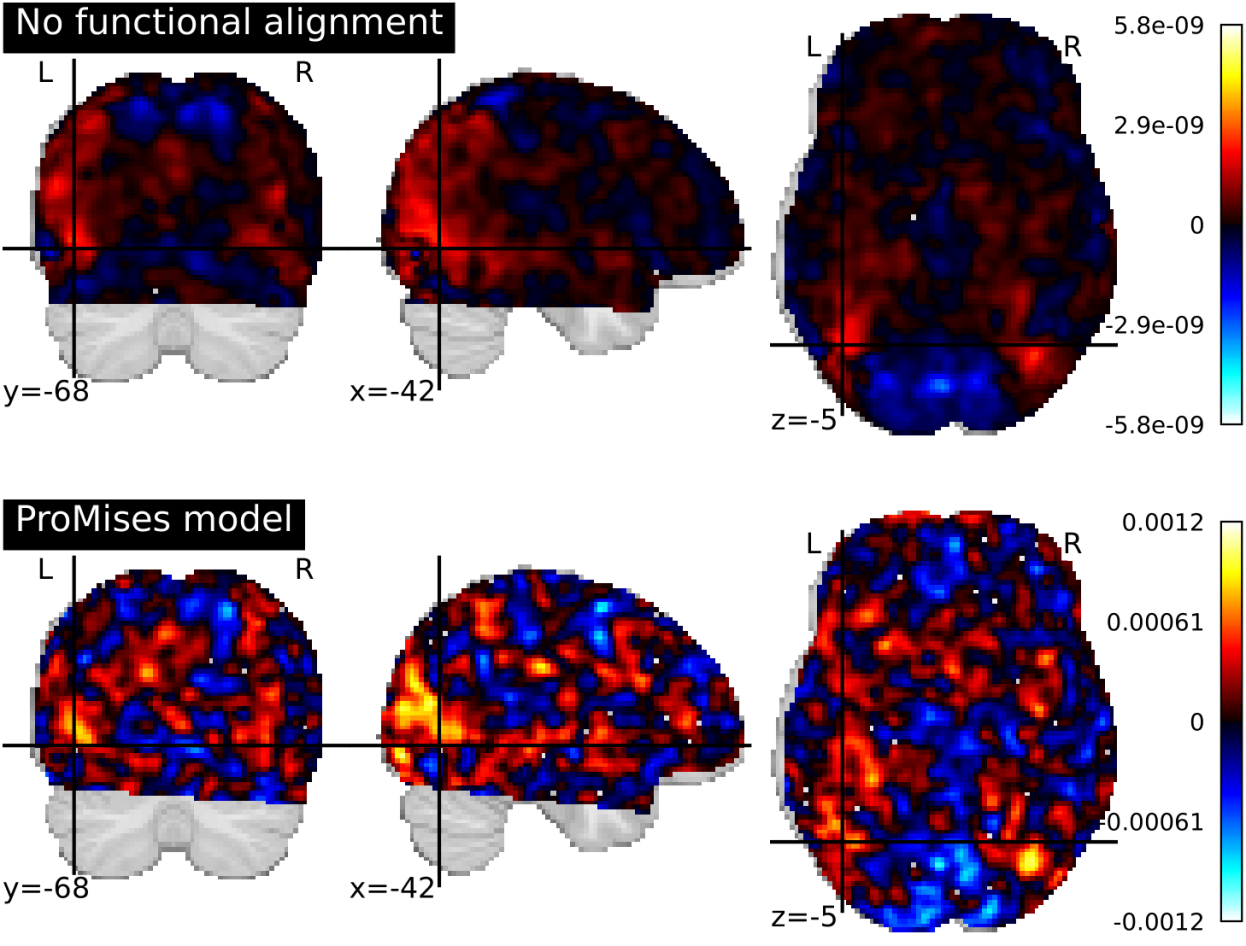
Coefficients of the multi‐class linear SVM considering the consonant string versus scrambled objects classifier (where hot colors correspond to predicting consonant string) analyzing data aligned via anatomical alignment and the ProMises model.

In this analysis, the entire brain is functionally aligned using the Efficient ProMises model in the same manner as described in the visual objects recognition data analysis. The brain images are then classified via SVM following the same procedure used for the faces and objects and visual objects recognition data sets. Figure 7 shows the coefficients of the consonant string versus scrambled objects classifier considering functionally aligned (bottom figure) and not functionally aligned (top figure) data. The between‐subject accuracy equals 0.2 if the data is not functionally aligned, while it is 0.33 if the data is functionally aligned. It is interesting to note the two yellow blobs which correspond to Brodmann area 19, which is known to be a visual processing area (Duncan et al., 2009; Wright et al., 2008), where heightened activation corresponds to predicting the consonant string. The analysis takes 6557.867 seconds if the Efficient ProMises model is used, in comparison it takes 1204.945 seconds if it is not used.

## 4 DISCUSSION

Functional alignment is a preprocessing step that improves the functional coherence of fMRI data, hence improving the accuracy of subsequent analysis (Guntupalli et al., 2016; Haxby et al., 2011; Haxby et al., 2020). This article presents a functional alignment method that solves most of the shortcomings present in previously used methods, particularly GPA (Gower, 1975) and hyperalignment (Haxby et al., 2011). The ProMises model returns aligned images that are interpretable, fully reproducible, and provide enhanced detection power. Below we summarize several of the key findings of this article.

### 4.1 Enhanced detection power

We applied the proposed method to four different data sets, allowing us to evaluate its performance under a number of different settings. This included differences in sample size, length of time series, whether data was extracted from an ROI or the whole‐brain, and whether the data resides in volumetric space or on the surface.

We further contrasted the approach with three other approaches. The first was simply performing no functional alignment (i.e., anatomical alignment only). The second, was the standard hyperalignment approach. The third, was the classic GPA approach. For the two latter approaches and ProMises anatomical alignment was performed prior to functional alignment.

For all four data sets the ProMises model greatly outperformed using no functional alignment, for example, see Table 2. Consistently for all settings the classification accuracy was roughly doubled when using the ProMises model in addition to standard anatomical alignment. Further, as can be seen in Figure 6, in most cases, the ProMises model outperformed the other functional alignment techniques in all permutations of these approaches. On occasion certain permutations outperformed ProMises, though this was rare.

A potential limitation of the study is that the performance of the anatomical‐only alignment is likely partially due to the spatial normalization procedure used. In this work, we applied AFNI's default approach, and it is possible that another approach would have given slightly different results. However, we do not anticipate this would have changed our conclusions.

### 4.2 Reproducibility and interpretability of the results

The solution (i.e., the final image) produced by GPA is not unique, as it depends on the starting point of the iterative algorithm. A similar issue arises for the hyperalignment method where the solution depends on the order in which subjects are entered into the algorithm. As a consequence, results may vary widely depending on arbitrary choices made by the experimenter (or the software used). The severity of this problem is visible in all analyses performed in this article. As an example, see Figure 6, where the accuracy is not given by a single value, but rather represented using a box‐and‐whisker plot. To the best of our knowledge, the ProMises model is the only Procrustes‐based functional alignment technique that resolves the problem of nonuniqueness of the solutions, thereby enhancing the reproducibility of the results.

The nonuniqueness of the solution further leads to difficulties with interpretability, since equivalent solutions in mathematical terms (i.e., where the same maximum is obtained) may provide different—sometime very different—final images. This reduces the previous alignment methods to black box solutions that do not directly improve our understanding of the underlying cognitive activities.

The ProMises model offers a way to address these two issues thanks to the inclusion of prior (and anatomical) information into the analysis. This makes the solution unique (i.e., reproducible), and the resulting images interpretable. The proposed solution borrows information from the whole brain, but is driven to act locally, therefore making the results anatomically meaningful. This can clearly be seen in Figure 5, which reports the contribution of a given voxel in the construction of the new, aligned, voxel as a function of the Euclidean distance. It is evident that the highest contribution comes from the voxels that are closest in proximity.

As confirmation of the interpretative quality of the method, we can study the map of classifier coefficients presented in Figure 4. The image is clear and interpretable if the functionally aligned fMRI data are used. For example, we can see a yellow blob of activation in Figure 4 in the functionally aligned data. The blob corresponds to the superior temporal gyrus, a region known to be involved in the perception of emotions in reaction to facial stimuli (Haxby et al., 2001; Ishai et al., 2000; Ishai et al., 1999). While, we can comfortably interpret these maps when using the ProMises model, it is more ambiguous for other methods. In fact, the other methods do not return a single aligned image; the representation of the results on the anatomical template is possible but without guarantee of validity from a mathematical point of view. We stress here that the classifier weight coefficients must be transformed to proper activation patterns (Haufe et al., 2014) if inferential conclusions are desired.

### 4.3 Computationally efficiency

While the proposed method is iterative, it is usually less computationally intensive than GPA (i.e., the nonregularized counterpart). The reason is that it typically reaches the convergence criteria (i.e., the Frobenius distance between the references matrices of two consecutive iterations is minimal [i.e., less than 0.001]) in only a few iterations thanks to the regularization term defined by the prior parameters (i.e., k and F). As an example, consider the first analysis in Subsection 3.3 (i.e., using the VT mask from the raiders data set). Here, the ProMises model takes 7581.615 s to perform the analysis, whereas GPA takes 48847.36 seconds using a 3000+ core Linux cluster with 20 GB of random‐access memory and parallel computation for the subjects (i.e., the analysis are parallelized across a number of cores equal to the number of subjects included in each analysis). hyperalignment only takes 2728.47 s, but it is does not reach any optimality criterion, as seen in Section 2.1. Finally, the computation time could be improved by using different approaches than cross‐validation (e.g., generalized cross validation; Golub & Von Matt, 1997 or bandwidth selection techniques; Heidenreich et al., 2013).

### 4.4 Whole brain applicability

More relevantly, the efficient extension of the ProMises model overcomes computational difficulties related to performing whole‐brain analysis that plague both hyperalignment and GPA. This extension of the model works on a reduced space of the data, thereby gaining in efficiency. In practice, the dimensions are reduced from the number of voxels to the number of scans, which for typical fMRI data implies a significant dimension reduction. A competing model in this context is searchlight hyperalignment (Guntupalli et al., 2016), where overlapping transformations are calculated for overlapping searchlights in each subject and then aggregated into a single whole‐brain transformation. While this allows for an anatomical interpretation of the final map, the final transformation is not an orthogonal matrix, and therefore will not preserve the content of the original data. While searchlight hyperalignment uses local radial constraints, the ProMises model incorporates them directly into the Procrustes estimation process through the prior, thus providing increased flexibility. Another approach is piecewise functional alignment (Bazeille et al., 2021), where nonoverlapping regions (coming from a priori functional atlas or parcellation methods) are aligned and then aggregated. Bazeille et al. (2021) found substantial improvement compared to using searchlight approaches in whole brain analysis. However, it suffers from possible staircase effects along the boundaries of the nonoverlapping regions.

### 4.5 Extensions

Because the proposed approach is a statistical model, various extensions can be considered to include more flexibility (e.g., examining subpopulations using different reference or location matrices). The specification of location matrix F as a similarity matrix also permits exploring various types of distances (e.g., considering the gyrus instead of the voxels as units). To conclude, the definition of F opens up a universe of different possibilities to express anatomical and functional constraints existing between voxels/regions in the brain. It is plausible that other functional alignment methods proposed in the literature (e.g., SRM proposed by Chen et al. (2015)) can be incorporated into the ProMises model, which can be explored in future work.

## 5 CONCLUSION

Together, these findings lead us to believe that the ProMises algorithm provides a promising approach toward performing functional alignment on fMRI data that improves classification accuracy across a number of different settings. We therefore believe it is an attractive option for performing functional alignment on the fMRI data prior to fitting predictive models.

## AUTHOR CONTRIBUTIONS

Angela Andreella: Conceptualization, methodology, formal analysis, and writing of the original draft. Livio Finos: Conceptualization, methodology, writing the original draft, and supervision. Martin A. Lindquist: Conceptualization, writing the original draft, and supervision.

## FUNDING INFORMATION

Angela Andreella gratefully acknowledges funding from the grant BIRD2020/SCAR_ASEGNIBIRD2020_01 of the Università degli Studi di Padova, Italy, and PON 2014‐2020/DM 1062 of the Ca' Foscari University of Venice, Italy. Martin A Lindquist was supported in part by NIH grants R01 EB016061 and R01 EB026549 from the National Institute of Biomedical Imaging and Bioengineering.

## CONFLICT OF INTEREST

The authors declare no competing interests.

## Figures and Tables

**FIGURE B1:**
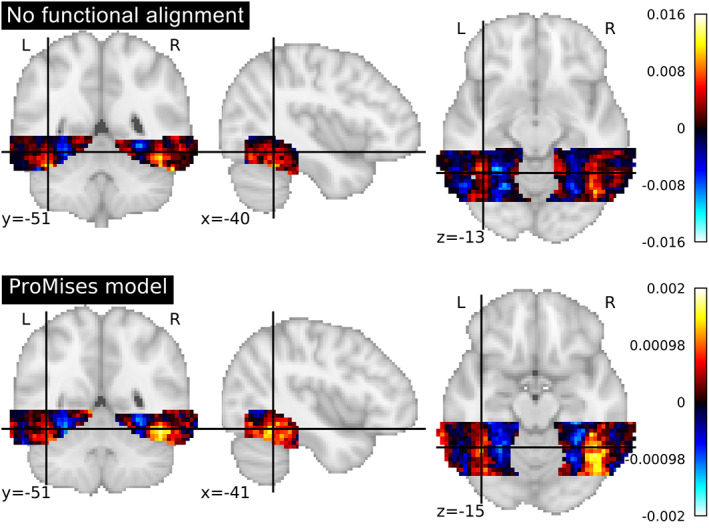
Coefficients of the multi‐class linear SVM considering the chair versus shoe classifier (where hot colors correspond to predicting chair) analyzing data aligned via anatomical alignment and the ProMises model.

**FIGURE B2:**
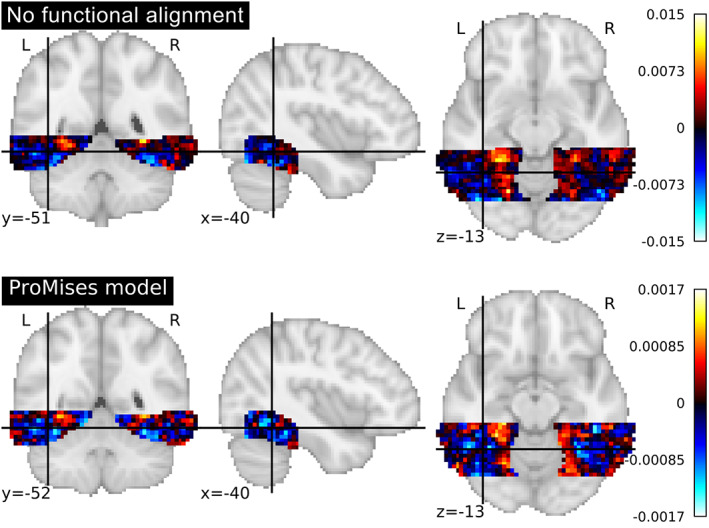
Coefficients of the multi‐class linear SVM considering the dog face versus shoe classifier (where hot colors correspond to predicting dog face) analyzing data aligned via anatomical alignment and the ProMises model.

## Data Availability

The faces and objects dataset can be downloaded from https://github.com/angeella/ProMisesModel/tree/master/Data/Faces_Objects, while the visual object recognition dataset from https://www.openfmri.org/dataset/ds000105/. The *raiders* dataset is currently not available online, but data can be made available upon request. However, a sub‐sample of 11 subjects can be found at DataLad: http://datasets.datalad.org/?dir=/labs/haxby/raiders. Finally, the *words and objects* dataset can be downloaded from https://www.openfmri.org/dataset/ds000107/.

## APPENDIX 1

### A ALGORITHMS

Algorithm A provides the pseudocode for the estimation process of the ProMises model, while Algorithm A shows the adaptation to perform its efficient version

## APPENDIX 2

### B FACES AND OBJECTS RESULTS

Figure B.1 represents the coefficients considering a classification of fine‐grained distinction among object categories (i.e., shoes versus a chair). In the same way, Figure B.2 represents the case of coarse‐grained distinctions (i.e., dog face versus shoe). Thanks to the model formulation of the proposed method, the coefficients of the classifiers can be represented in brain space, returning maps with spatial boundaries between different categories of stimuli.
